# Ileosigmoid knot in a patient with Down syndrome: a unique surgical emergency

**DOI:** 10.11604/pamj.2021.38.8.27407

**Published:** 2021-01-05

**Authors:** Said Boussaidane, Abderrahim Samlali, Asma Hamri, Youssef Narjis, Benomar Ridouan Benelkhaiat

**Affiliations:** 1 Department of General Surgery, Ibn Tofail Hospital, Mohamed VI University Hospital, Cadi Ayad University, Marrakech, Morocco

**Keywords:** Ileosigmoid knot, volvulus, bowel obstruction, necrosis, Down syndrome, case report

## Abstract

The ileosigmoid knot (ISK) or double ileosigmoid volvulus is a wrapping of the small intestine around the base of the sigmoid colon. We report an unusual case in the digestive surgery department of the Ibn Tofail Hospital of CHU Mohammed VI Marrakech of a 28-year-old man with Down's syndrome who presented with symptoms and signs of intestinal obstruction. Abdominal CT scan revealed a whirl sing and significant distension of the sigmoid loop. Exploratory laparotomy revealed ISK resulting in gangrene of ileum and sigmoid colon. The surgical procedure was a necrotic digestive segments resection, with a double-barrelled ileostomy and a Hartmann procedure. One month afterwards, the patient was operated on to reestablish of the continuity. Through this observation and a review of the literature we define the diagnostic, therapeutic and prognosis aspects of this rare clinical entity.

## Introduction

The ileosigmoid knot (ISK) or double ileosigmoid volvulus is uncommon cause of acute intestinal obstruction, it is a wrapping of the small intestine around the base of the sigmoid colon [1], thus achieving intestinal obstruction by bifocal strangulation of the sigmoid and ileum [2]. The ISK is considered to be a true surgical emergency which rapidly progresses to intestinal necrosis. The pre-operative diagnosis is difficult. Knowledge of the mechanism of this pathology is essential, allows early and appropriate diagnosis and management [3].

## Patient and observation

**Figure 1:**
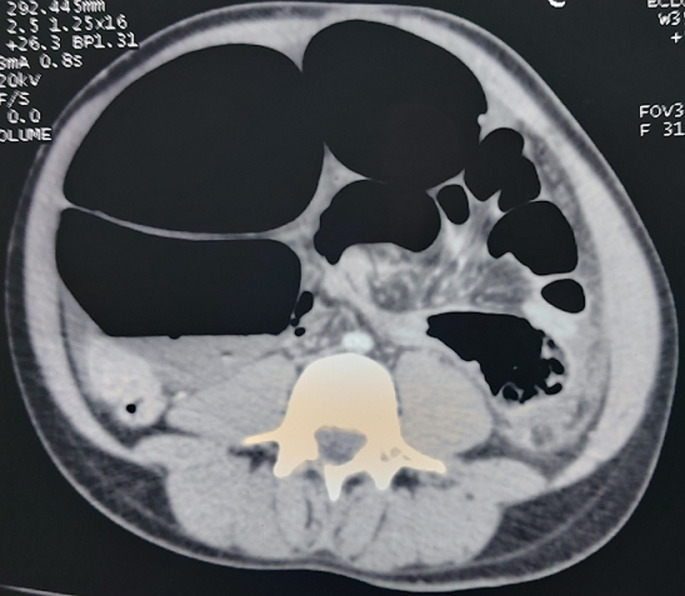
the abdominal CT scan revealed a whirl sing and significant distension of the sigmoid loop, measuring 12 cm in diameter with parietal thinning

A 28-year-old man with Down's syndrome, operated on in childhood for interatrial communication, admitted to the emergency department for intense abdominal pain with vomiting and obstipation, evolving for 3 days. On admission, the patient was anxious, asthenic with polypnea and tachycardia (heart rate of 151 beats per minute), blood pressure of 70/50 mmHg. The abdomen distended, tender and tympanic on palpation, free hernial orifices and empty ampulla on digital rectal examination. Blood tests revealed a level of plasma C-reactive protein increased to 106.3 mg/l, functional renal insufficiency with creatinine of 23.8 mg/l and bicarbonate of 17.6 mmol/l. Abdominal X-ray showed significant distension of the large bowel, coffee-bean sign, with air-fluid levels. Abdominal computed tomography (CT) scan: a whirl sing and significant distension of the sigmoid loop, measuring 12 cm in diameter with parietal thinning (Figure 1).

**Figure 2:**
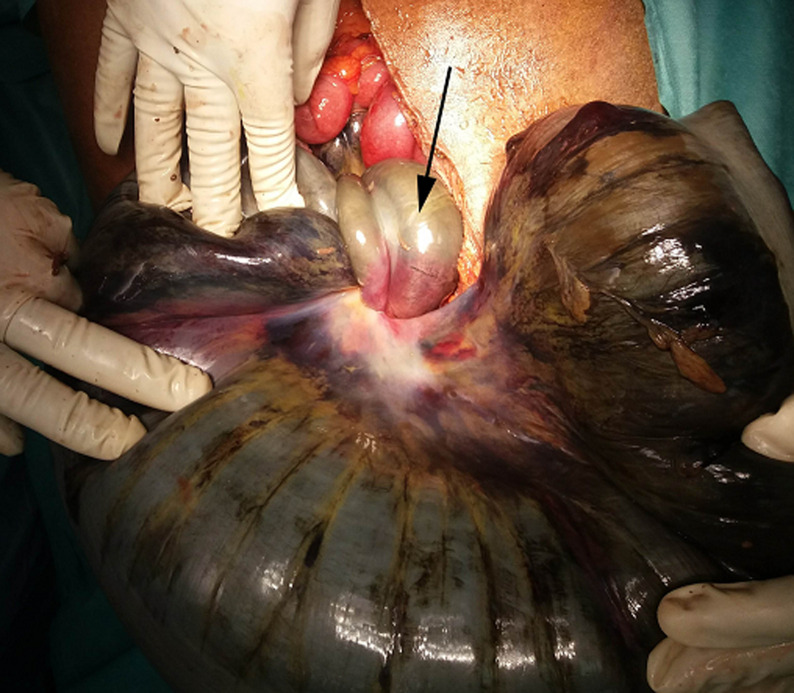
intraoperative view of the ileal node (arrow) around the base of the sigmoid, with necrosis of the twisted segments

**Figure 3:**
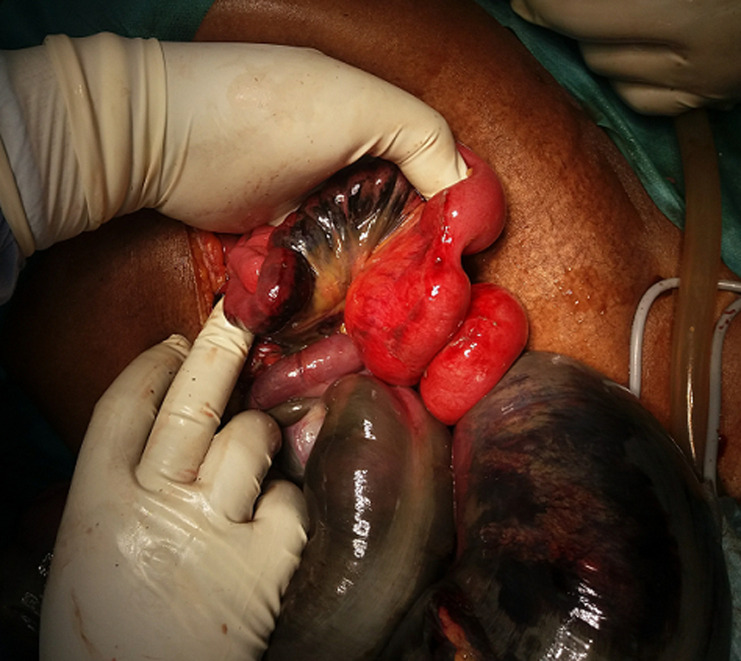
intraoperative view showing digestive necrosis after knot detorsion

An urgent midline laparotomy was performed after hemodynamic correction, surgical exploration had found low abundance ascites fluid, a enorm distension with necrosis of the sigmoid loop, the small bowel wrapping around the base of the sigmoid colon, making an ileosigmoid node, with necrosis of this torded ileum over 9 cm at 20 cm from the ileocecal junction (Figure 2, Figure 3). The surgical procedure was a necrotic digestive segments resection, with a double-barrelled ileostomy on the right and a Hartmann-type left colostomy. The patient was hospitalized postoperatively in the intensive care unit for 12 hours. One month afterwards, the patient was operated on to re-establish the small bowel and colonic continuity.

## Discussion

The ISK or double ileosigmoid volvulus, corresponds to the simultaneous strangulation of the ileum and the sigmoid colon. A rare pathology, which mainly affects men in the fourth decade [2]. Our case of ISK in a young man with Down's syndrome makes for a rare diagnosis. Atamanalp [4] suggested that high altitude, high-fiber diet habits, male gender and advanced age are known as probable causes of an anatomical predisposition to ISK, namely, an elongated sigmoid colon with a narrow mesentery in addition to a hypermobile terminal ileum. In our case, we can suggest the presence of congenital mega-colon which is part of the malformations of the digestive tract in Down's syndrome as an anatomical cause of ISK [5].

ISK has been classified into four subtypes, however, the exact mechanism of node development remains unclear. Type I (53.9 to 57.5%) is the most common, where the ileum (active component) wraps around the sigmoid colon (passive component). Type II (18.9-20.6%) is the opposite, where the sigmoid colon acts as an active component. Type III (1.5%) is the ileocecal junction which wraps around the sigmoid loop. And type IV is an indeterminate type, it is not possible to differentiate the two segments [6].

The preoperative diagnosis is difficult because of its rarity and clinico-radiological atypia, it is possible in less than 20% of cases [3]. Abdominal pain is constant, often sub-acute and progressive, associated with obstipation (98.8-100%), abdominal distension (94-100%), nausea and vomiting (77.5-100%) and rebound tenderness (36.8-100%) [4,6,7]. Abdominal X-ray may show disproportionate distension with large air-fluid levels in the sigmoid colon occupying the right side of the abdomen, with multiple air-fluid levels of the small intestine on the left side of the abdomen [3]. The abdominal CT scan can aid in the diagnosis by showing the whirl sign, created by the twisted meso-colon, mesenteric vessels and intestines [7]. CT can detect medial deviation of the distal descending colon with a pointed appearance of its medial border, which is a distinct feature of the ISK. Even though the cecum may be deviated medially in many patients, the pointed appearance of its medial border concurrent with the medial deviation of the distal descending colon are helpful features in diagnosing an ISK [8].

After an early and effective resuscitation, an emergency laparotomy is needed. When the intestine is viable, detorsion alone can be used, sigmoid mesopexy or mesoplasty can be added, whereas sigmoid resection may be performed to prevent volvulus recurrence [4]. In the event of intestinal necrosis, resection of the small bowel, colon and node in one piece is recommended [3], with primary anastomosis if local and general conditions allow it, otherwise a left iliac colostomy or outright a double segment stoma [4]. ISK is potentially lethal condition, with a mean mortality rate of 35.5% [6]. The most common cause of death being septic shock leading to multiple organ failure. Factors associated with mortality include advanced age, duration of symptoms (>24 hours since the onset of symptoms) and the extent of gangrene [7].

## Conclusion

The ISK is a rare cause of acute intestinal obstruction, which progresses rapidly to digestive necrosis, its preoperative diagnosis is difficult, only rapid surgical management can improve the prognosis of this pathology.
